# Anti-Edematogenic and Anti-Granuloma Activity of a Synthetic Curcuminoid Analog, 5-(3,4-Dihydroxyphenyl)-3-hydroxy-1-(2-hydroxyphenyl)penta-2,4-dien-1-one, in Mouse Models of Inflammation

**DOI:** 10.3390/molecules24142614

**Published:** 2019-07-18

**Authors:** Nadia Hisamuddin, Wan Mastura Shaik Mossadeq, Mohd Roslan Sulaiman, Faridah Abas, Sze Wei Leong, Nadhirah Kamarudin, Hui Ming Ong, Ahmad Farhan Ahmad Azmi, Rasyidah Ryta Ayumi, Madihah Talib

**Affiliations:** 1 Department of Veterinary Preclinical Sciences, Faculty of Veterinary Medicine, Universiti Putra Malaysia, Serdang 43400, Selangor, Malaysia; 2 Department of Biomedical Sciences Faculty of Medicine and Health Sciences, Universiti Putra Malaysia, Serdang 43400, Selangor, Malaysia; 3 Department of Food Science, Faculty of Food Science and Technology, Universiti Putra Malaysia, Serdang 43400, Selangor, Malaysia; 4 Laboratory of Natural Products, Institute of Bioscience, Universiti Putra Malaysia, Serdang 43400, Selangor, Malaysia; 5 Department of Microbiology, Faculty of Biotechnology and Biomolecular Sciences, Universiti Putra Malaysia, Serdang 43400, Selangor, Malaysia

**Keywords:** curcuminoid, synthetic analog, anti-inflammatory, edema, granuloma

## Abstract

Curcumin, derived from the rhizome *Curcuma longa*, has been scientifically proven to possess anti-inflammatory activity but is of limited clinical and veterinary use owing to its low bioavailability and poor solubility. Hence, analogs of curcuminoids with improved biological properties have been synthesized to overcome these limitations. This study aims to provide the pharmacological basis for the use of 5-(3,4-dihydroxyphenyl)-3-hydroxy-1-(2-hydroxyphenyl)penta-2,4-dien-1-one (DHHPD), a synthetic curcuminoid analog, as an anti-edematogenic and anti-granuloma agent. The carrageenan-induced paw edema and the cotton pellet-induced granuloma assays were used to assess the anti-inflammatory activity of DHHPD in mice. The effects of DHHPD on the histaminergic, serotonergic, and bradykininergic systems were determined by the histamine-, serotonin-, and bradykinin-induced paw edema tests, respectively. DHHPD (0.1, 0.3, 1, and 3 mg/kg, intraperitoneal) evoked significant reductions (*p* < 0.05) in carrageenan-induced paw edema at different time intervals and granuloma formation (*p* < 0.0001) by 22.08, 32.57, 37.20, and 49.25%, respectively. Furthermore, DHHPD significantly reduced paw edema (*p* < 0.05) induced by histamine, serotonin, and bradykinin. The present study suggests that DHHPD exerts anti-edematogenic activity, possibly by inhibiting the synthesis or release of autacoid mediators of inflammation through the histaminergic, serotonergic, and bradykininergic systems. The anti-granuloma effect may be attributed to the suppression of transudative, exudative, and proliferative activities associated with inflammation.

## 1. Introduction

Long-term regular use of commercially available non-steroidal anti-inflammatory drugs (NSAIDs) for the management of inflammation and pain can induce gastro-duodenal ulceration, abdominal pain, dyspeptic symptoms, hemorrhage, and perforation of the gastric organs, possibly resulting in hospitalization and even death [1]. This side-effect profile of NSAIDs has prompted researchers to seek alternatives in the form of extracts and compounds derived from naturally occurring medicinal plants, rhizomes, or herbs that are available all year round, abundantly found in nature, and affordable to low-income groups.

Rhizomes such as turmeric have been used as alternative anti-inflammatory agents in folklore medicine for decades [2,3]. Turmeric or *Curcuma longa* (*C. longa*) consists of three main bioactive constituents: curcumin, bisdemethoxycurcumin, and demethoxycurcumin. Collectively, these are referred to as curcuminoids [4]. Curcumin, which is the major constituent of turmeric, is considered to be responsible for the rhizome’s biological activity [5].

Curcumin exhibits anti-carcinogenic [6], anti-microbial [4], and antioxidant activities [7], and is safe for use even at high doses [8]. Curcumin has been shown to exert its in vivo anti-granuloma activity by augmenting the anti-inflammatory activities of cyclooxygenase inhibitors, such as aspirin and rofecoxib [9]. Despite these benefits, curcumin suffers from low bioavailability and poor solubility [4], thus limiting its medicinal and therapeutic potential in human and veterinary medicine. In order to overcome these limitations, various curcumin analogs with improved bioavailability, permeability, and solubility profiles have been synthesized and evaluated for various pharmacological applications [10,11]. Previously, Leong et al. [12] synthesized a series of diarylpentanoid derivatives and assessed their anti-inflammatory effects in vitro. Their results showed that two diarylpentanoid derivatives, referred to as Compound 88 (C88) and Compound 97(C97), both produced significant suppression of nitric oxide (NO) activity in the interferon-γ/lipopolysaccharide-stimulated RAW 264.7 macrophages. Additionally, both compounds exhibited more potent anti-inflammatory activity in vitro than curcumin and other diarylpentanoid derivatives.

Despite its good chemical stability and promising human intestinal absorption (HIA), C88 was found to be hepatotoxic and may produce irritation to the eyes and sensitization of the skin [12]. Based on the results of absorption, distribution, metabolism, excretion, and toxicological analysis (ADMET) and toxicity prediction of compounds using computer-aided technology (TOPKAT) for C88 and C97 reported by Leong et al. [12], C97 or 5-(3,4-dihydroxyphenyl)-3-hydroxy-1-(2-hydroxyphenyl)penta-2,4-dien-1-one (DHHPD) was selected for this study as it is non-hepatotoxic, does not show skin-sensitizing or ocular irritating properties, possesses good HIA, and, most importantly, it is highly soluble in water as compared to other synthetic curcuminoid analogs. Moreover, the presence of a hydroxyl group on both of its aromatic rings renders it more soluble than the Cl^−^-bound C88. In spite of its good bioavailability and in vitro activities, C88 and other compounds containing the Cl^−^ group are potentially unsafe for animal and human consumption or for use as a medication since excessive or chronic Cl^−^ intake and use exerts toxic effects in plants [13], humans, and animals [14]. In this work, we report the anti-inflammatory effects of DHHPD in vivo and the possible mechanisms that are responsible for its pharmacological activities.

## 2. Results

### 2.1. Acute Toxicity Study

The pretreated mice did not show any respiratory distress, diarrhea, sedation, or motor impairment during the initial 3 h observation period. No mortalities were recorded over the 7-day test period and the vital organs were normal upon post mortem examination. The results of the acute toxicity study for DHHPD corroborates the findings reported by Kamarudin et al. [15].

### 2.2. Anti-Inflammatory Study

#### 2.2.1. Carrageenan-Induced Paw Edema Test

At 1 h post-induction, DHHPD at doses of 0.3, 1, and 3 mg/kg (intraperitoneally, i.p.) significantly (*p* < 0.05) decreased paw edema formation by 20.00%, 25.26%, and 36.84%, respectively (Table 1). At these doses, DHHPD continued to produce a significant (*p* < 0.0001) and sustained increase in the inhibition of inflammation until the end of the experiment (5 h), demonstrated by inhibition levels of 66.92%, 73.85%, and 86.92%, respectively. With respect to DHHPD (0.1 mg/kg, i.p.) and acetylsalicylic acid (ASA) (100 mg/kg, i.p.), a significant reduction in edema was observed from 2 h post-induction onwards. The inhibition produced by DHHPD (3 mg/kg, i.p.) at 2 h, 3 h, 4 h, and 5 h post-induction (57.94%, 69.23%, 78.33%, and 86.92%, respectively) was greater than that induced by ASA (35.51%, 50.43%, 60.83%, and 69.23%, respectively) or DHHPD at lower doses (0.1, 0.3, and 1 mg/kg, i.p.). Moreover, DHHPD (3 mg/kg, i.p.) significantly reduced the paw edema 5 h after induction, at a measurement approaching the basal thickness of the paw edema at 0 h. In this model, the calculated ED_50_ value for DHHPD was 1.11 mg/kg, i.p. (Confidence interval, or CI, 0.81 to 1.54 mg/kg).

#### 2.2.2. Cotton Pellet-Induced Granuloma Test

DHHPD at 0.1, 0.3, 1, and 3 mg/kg (i.p.) significantly (*p* < 0.0001) decreased granuloma formation by 22.08%, 32.57%, 37.20%, and 49.25%, respectively (Table 2). The 49.25% inhibition induced by the maximum dose of DHHPD (3 mg/kg, i.p.) was comparable to that observed for ASA (49.70%). For this test, the calculated ED_50_ value for DHHPD was 0.59 mg/kg, i.p. (CI, 0.15 to 2.43 mg/kg).

### 2.3. Involvement of the Histaminergic, Serotonergic and Bradykininergic System

#### 2.3.1. Histamine-Induced Paw Edema Test

**Figure 1:**
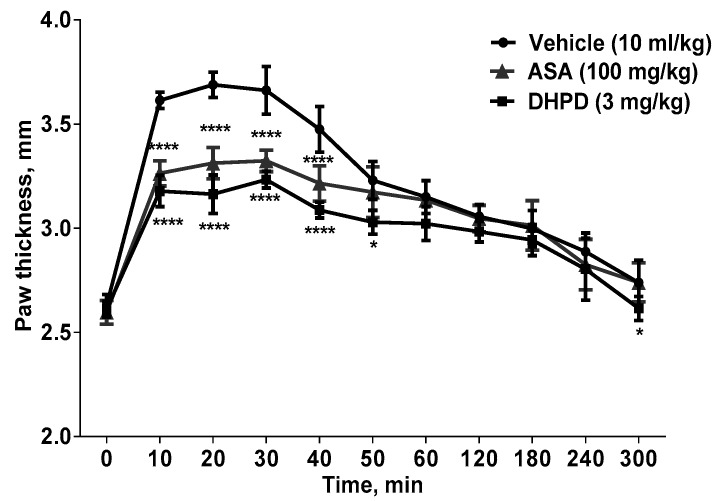
Effects of DHHPD on histamine-induced paw edema in mice (*n* = 6). The x-axis represents the interval (min) after histamine injection. * *p* < 0.05 and **** *p* < 0.0001 compared to vehicle (two-way ANOVA followed by Dunnett’s post hoc test).

In the present histamine-induced paw edema study (Figure 1), results showed that the formation of paw edema was significantly (*p* < 0.0001) inhibited by an intraperitoneal administration of DHHPD (3 mg/kg) beginning from the 10th min until the 50th min post histamine injection.

#### 2.3.2. Serotonin-Induced Paw Edema Test

**Figure 2:**
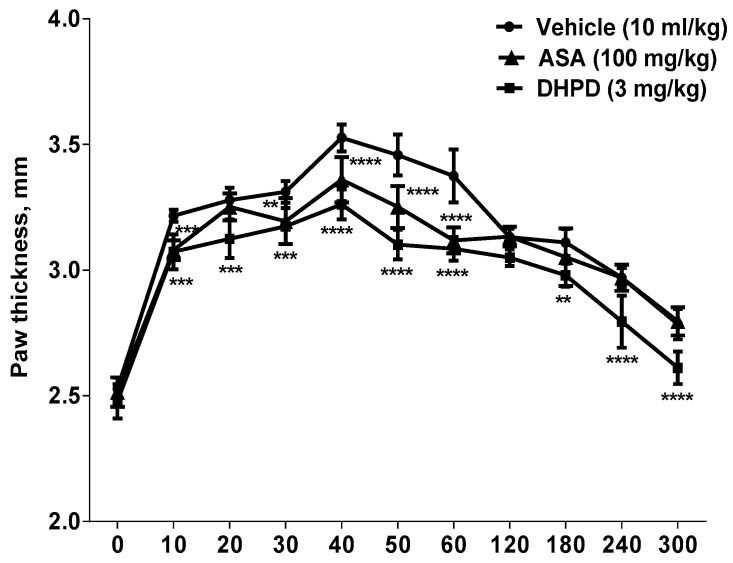
Effects of DHHPD on serotonin-induced paw edema (*n* = 6). The x-axis represents the interval (min) after serotonin injection. ** *p* < 0.01, *** *p* < 0.001 and **** *p* < 0.0001 compared to vehicle (two-way ANOVA followed by Dunnett’s post hoc test).

In the serotonin-induced paw edema test (Figure 2), DHHPD (3 mg/kg, i.p.) significantly inhibited the formation of paw edema (*p* < 0.01) at the first hour and from the third to fifth hour.

#### 2.3.3. Bradykinin-Induced Paw Edema Test

**Figure 3:**
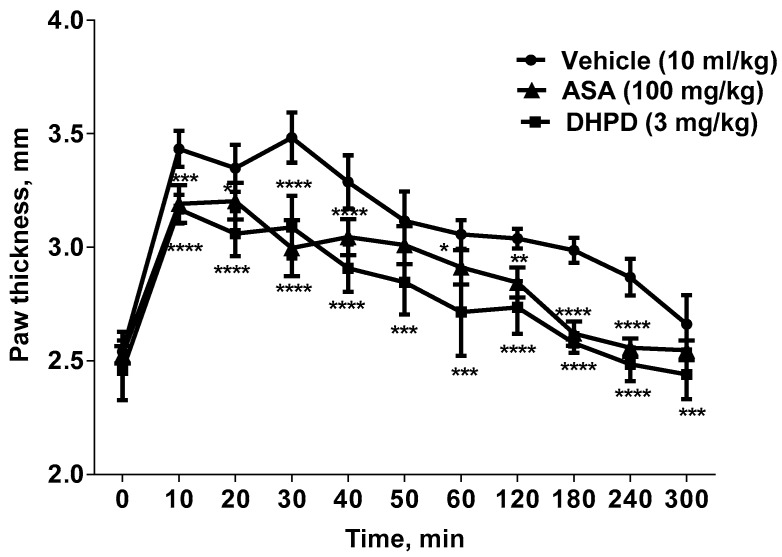
Effects of DHHPD on bradykinin-induced paw edema (n = 6). The x-axis represents the interval (min) after bradykinin injection. * *p* < 0.05, ** *p* < 0.01, *** *p* < 0.001 and **** *p* < 0.0001 compared to vehicle (two-way ANOVA followed by Dunnett’s post hoc test).

The results obtained from the bradykinin-induced paw edema test (Figure 3) showed that paw edema formation was significantly (*p* < 0.01) and consistently reduced by DHHPD (3 mg/kg, i.p.) throughout the experiment (i.e., from the first until the fifth hour of the experiment).

## 3. Discussion

NSAIDs have long been the most popular choice for immediate treatment of inflammatory conditions. However, their frequent use can be accompanied by serious side effects, thus prompting researchers, practitioners of traditional medicine, and patients to seek alternatives in the form of herbs, rhizomes, and wild plants with anti-inflammatory properties. In this study, we investigated the effects of DHHPD, a synthetic diarylpentanoid curcuminoid analog, on carrageenan-induced paw edema. The results showed that DHHPD (3 mg/kg, i.p.) significantly attenuated the paw edema induced by carrageenan, indicating probable suppression of the release and/or synthesis of inflammatory mediators during the acute stage of inflammation.

The carrageenan-induced paw edema model is frequently used in the evaluation of the acute anti-inflammatory properties of novel products owing to its high reproducibility [16]. This model produces a biphasic event, firstly mediated by the release of histamine, serotonin, and 5-hydroxytryptamine from 0 to 2 h after carrageenan injection (first phase) [17], followed by the bradykinin-mediated release of kinins and prostaglandin-like substances [18] and increased cyclooxygenase activity from 3 to 5 h post-carrageenan injection (second phase) [19].

Upon tissue injury or in response to stress or an immunological trigger, histamine stored in the basophilic granules and platelets is released into the bloodstream, concurrent with mast cell degranulation. The release of histamine increases blood flow and vascular permeability at the injured area, leading to the leakage of fluid and proteins from the blood into the spaces between the tissues. These events occur during the first phase of the carrageenan test. However, antihistamine drugs commonly prescribed for allergies and many forms of inflammation may affect these conditions by competing for histamine receptors, thereby preventing histamine attachment and consequently reducing inflammation and its associated clinical signs [20]. DHHPD (3 mg/kg, i.p.) significantly reduces the formation of edema induced by intraplantar administration of histamine, suggesting that DHHPD suppresses the release of histamine from mast cells, basophils, or platelets during the acute stage, possibly through antagonism of histamine receptors.

Bradykinin, an autacoid mediator that is released 2 h post injury, induces vasodilation, smooth muscle contraction, and pain during inflammation [21]. The B1 receptors that are present on peripheral afferent nerves play a role in exacerbating the inflammatory response [22]. In our study, DHHPD (3 mg/kg, i.p.) significantly reduced carrageenan- and bradykinin-induced paw edema, suggesting that DHHPD inhibits the release of bradykinin, which mediates the first and second phases of the model through the antagonism of bradykinin receptors on the peripheral nerves, thus reducing the severity of the inflammation produced.

It is known that during an inflammatory event, bradykinin stimulates phospholipase to produce and release prostaglandins into the system towards the end of the second phase and during the third phase of the carrageenan-induced paw edema test [23]. Prostaglandins are also produced by tissue macrophages after injury, along with leukotrienes and polymorphonuclear cells, causing vasodilation, in addition to pain and pyrexia. The dramatic increase in the level of circulating prostaglandins during inflammation is the result of up regulation of inducible cyclooxygenase-2 (COX-2) on the surface of activated macrophages, often occurring within several hours of an injury [24]. In addition to the COX enzyme, lipoxygenases (LOX), which are readily expressed in leucocytes and various tissues, such as healthy epithelial and immune cells, as well as non-healthy apoptotic cells, play a significant role in the inflammatory process through their involvement in the regio- and stereo-specific peroxidation of arachidonic acid and linoleic acid [25,26]. It is well established that NSAIDs reduce inflammation and pain by inhibiting the synthesis of prostaglandin and the enzymatic activity and gene expression of COX-1 and COX-2 in inflamed tissues [27,28,29]. However, it is also known that dietary curcumin exhibits chemopreventive activity against colonic tumors by inhibiting phospholipase A2, altering the cyclooxygenase and lipoxygenase activities and simultaneously modifying the levels of PGE_2_ [30], prostaglandin D_2_, and 6-ketoprostaglandin F_1α_ [31]. Moreover, curcumin evidently decreases the expression of COX-2 in phorbol ester- and bile acid-challenged human gastrointestinal epithelial cells at the transcriptional level [32], and reduces the production of LOX metabolites and LOX activity in colonic mucosa and tumor cells [33,34]. In our study, the administration of DHHPD (3 mg/kg, i.p.) significantly inhibited paw edema in both phases of the test, while ASA (100 mg/kg, i.p.), which was used as the reference drug, only caused significant inhibition from 2 h onwards. In addition, DHHPD has been shown to reduce the frequency of abdominal constrictions in the 0.6% acetic acid test and the latency of paw licking in the 2.5% formalin test in mice, suggesting its peripheral mechanism in inhibiting the release of inflammatory mediators responsible for pain [15]. These results indicate that DHHPD may act on the COX enzymes or prostaglandin synthesis from arachidonic acid through a mechanism similar to that of aspirin. Thus, it appears that both curcumin and its synthetic analog act through a mechanism similar to that of aspirin in reducing the levels of COX and prostaglandins in circulation during inflammation.

Previously, LY53857 was reported as the most potent antagonist of serotonin-induced paw edema [35] Furthermore, dopamine agonists, such as quinpirole and pergolide, have been reported to exhibit significant antagonism of the serotonin-induced response; both drugs are similar to LY53857 in structure [35]. The effect produced by dopamine agonists on the serotonin-induced inflammatory response could be associated with the autonomic nervous system, which plays a vital role in regulating the blood flow to the periphery and pressure of perfusion in the vasculature [35]. In this work, DHHPD was shown to significantly reduce edema in the carrageenan- and serotonin-induced paw edema tests, suggesting the suppression of serotonin release activity following tissue trauma, indicating the possible role of DHHPD as a serotonin receptor antagonist. It is thus plausible that DHHPD is indirectly involved in controlling blood flow to the damaged tissues through activation of dopamine receptors and/or antagonism of serotonin receptors, thereby inhibiting the inflammatory response. Further investigation is required to gain a better understanding of this mechanism.

Tamaddonfard et al. [36] reported the possible involvement of curcumin in potentiating central activity in the paw edema test. Curcumin potentiates the effects of morphine on edema when administered together. However, the suppressive effect on edema produced by curcumin alone and by the curcumin–morphine combination was not antagonized by naloxone, indicating a non-opioid-dependent anti-edema effect. In addition, the analgesia induced by DHHPD in the mice models of induced anti-nociception did not involve the opioidergic system [15]. Thus, it appears that curcumin and its analog DHHPD both present anti-edema activity, possibly through a non-opioidergic mechanism.

The development of edema in carrageenan-instigated acute inflammation is also ascribed to the release of nitric oxide synthase (NOS) isoforms, such as endothelial, neuronal, and inducible NOS. The soluble form of NO in circulation acts as a potent vasodilator and, in combination with NOS, induces tissue damage and hyperalgesia. Regarding nitric oxide involvement, curcumin has been shown to inhibit NO production as well as the expression of inducible NOS protein and mRNA in LPS/interferon-γ-stimulated RAW 264.7 cells [37]. In addition, DHHPD (C97) has been reported to significantly attenuate NO expression in vitro [12], an effect possibly attributed to the presence of the catechol moiety of this compound. It is known that neuronal NOS is implicated in the first and second phases of inflammation, while inducible NOS plays a major role much later, during the second phase of inflammation. Our results showed that DHHPD reduces paw edema formation during the first and second phases of the carrageenan-induced paw edema test, suggesting that DHHPD also inhibits the neuronal and inducible forms of NO expression in vivo. Closer evaluation of the effects of DHHPD on various forms of NOS expression in vivo is recommended to support the above findings.

The anti-inflammatory effects of curcumin in its natural, semi-synthetic, and sodium salt forms in various in vivo and in vitro models of inflammation received a significant amount of attention in the 1970s and 1980s [38,39,40,41]. It has been suggested that the effectiveness of curcumin as an anti-inflammatory, antioxidant, and anti-cancer agent is attributed to its free radical scavenging activities, performed by its phenolic, methylenic, and β-diketo groups, in addition to its hydroxyl and methoxy substituents, which further enhance the effects produced [42,43,44]. However, its biological activities are limited by its rapid metabolism and insolubility, which render it ineffective at low doses.

A previous study showed that curcumin at an oral dose of 400 mg/kg exhibits anti-edema activity during the second phase of the carrageenan-induced paw edema test [45]. The findings are in contrast to those for DHHPD, which showed better anti-inflammatory activities in both phases of the test, even at lower doses. The lower activity demonstrated by curcumin may likely be due to its efficient first-pass metabolism and intestinal metabolism, resulting in low bioavailability in the blood circulation [46]. The low bioavailability and clinical efficiency of curcumin may also be accredited to its chemical instability under physiological conditions [47]. Conversely, the ability of DHHPD to produce an anti-inflammatory response during both phases of the test could be attributed to the existence of a hydroxyl group on both of its aromatic rings, resulting in it having higher circulatory bioavailability and solubility than curcumin. Moreover, ADMET analysis of DHHPD indicated its good aqueous solubility and HIA properties [12]. Liang et al. [47] had synthesized a series of curcumin analogs which was then evaluated for their chemical stability at different time points using the UV absorbance spectroscopy. Results from the study showed that the analogs were more chemically stable compared to curcumin, which underwent a significant rate of degradation. The results from the study suggested that a curcumin analog such as DHHPD may also exhibit similar in vitro chemical stability. However, the extent or efficiency of DHHPD metabolism in the gut in comparison to curcumin and other curcuminoid analogs in vivo and its in vitro chemical stability as well as its hematology profiles and are not known at present and are the focus of our current research.

The other factor that may contribute to the good anti-inflammatory activities exhibited by DHHPD is the route of administration employed in the study versus previous curcumin studies. The intraperitoneal/parenteral route of administration was chosen in this study for its systemic effect and its ability to maintain the highest bioavailability of substances in circulation by avoiding first-pass metabolism, which commonly occurs in enteral administration. Curcumin is readily metabolized and excreted by the gut. Therefore, intraperitoneal administration of DHHPD avoids some of the unpredictability often associated with enteral absorption processes. However, a comparative study on the effectiveness of DHHPD in inducing anti-inflammatory activities via different routes of administration is currently underway in our laboratory.

The cotton pellet-induced granuloma test is a conventional model for the chronic inflammatory response, through which the effects of certain substances on fluid extravasation, granuloma formation, and biochemical exudation can be readily detected. Inflammation and granuloma developing over several days following a sterilized cotton implant are indicative of the proliferative phase of inflammation involving the active participation of macrophages, neutrophils, and fibroblasts and activation of leukocytes, such as monocytes and lymphocytes [48]. In this test, monocyte-derived macrophages are released to the site of injury to eliminate the cotton pellet implant, which was earlier identified as a foreign substance by the animal’s biological system. However, when macrophages fail to eliminate the foreign object, IL-12 is released, followed by activation of T-lymphocytes, which release IFN-γ, thereby flooding the site of injury with additional macrophages. Such a massive release of macrophages and T-lymphocytes leads to the formation of a granuloma as the macrophages fuse with one another [48]. In this test, the extent of granulomatous tissue formed is correlated to the dried weight of the cotton pellets harvested, while the wet weight of cotton pellets is correlated with the presence of transudate.

NSAID-mediated reduction of granuloma size is the consequence of a cellular response involving the inhibition of granulocyte infiltration, the production of collagen fibers, and the suppression of mucopolysaccharides. Our results showed that the decrease in the weight of the dried granuloma in mice treated with DHHPD at all doses was comparable to that produced by ASA treatment. We can thus conclude that DHHPD effectively suppresses the proliferative phase, possibly via a similar mechanism to that of NSAIDs. Further mechanistic tests to assess the effects of DHHPD on macrophage-derived pro-inflammatory cytokines are required to substantiate the reasoning behind these findings.

Results from the in vivo acute toxicity study indicated that DHHPD (0.1, 0.3, 1, and 3 mg/kg, i.p.) did not induce any mortalities or abnormal behavior in mice. According to Burgos-Morón et al. [49], curcumin and its derivatives induces genotoxicity and high levels of toxicity in vitro and in human subjects, and cautioned its use in clinical practice and therapeutic interventions. In contrast, a significant body of research suggested that curcumin and/or its analog demonstrated significant antimutagenic activities [50,51,52,53,54] and inhibitory effect on genotoxicity induced by mutagens in cooked food [55]. However, the doses and concentrations of curcumin and its derivatives used in these studies vary. Hence, the probable antimutagenic activity, genotoxicity and chronic toxicity effects of DHHPD in comparison to curcumin and other analogs are needed to support the findings of the acute toxicity assay.

## 4. Materials and Methods

### 4.1. Synthesis, Structural Identification and Confirmation of 5-(3,4–Dihydroxyphenyl)-3-hydroxy-1-(2-hydroxyphenyl)penta-2,4-dien-1 One

**Figure 4:**
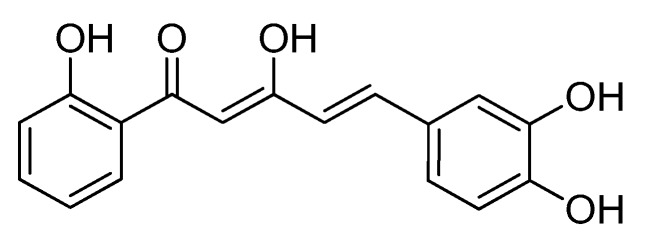
Chemical structure of DHHPD.

Synthesis of DHHPD was performed by Associate Professor Dr. Faridah Abas and colleagues at the Laboratory of Natural Products, Institute of Bioscience, Universiti Putra Malaysia (IBS, UPM). The desired DHHPD (Figure 4) was synthesized via a Knoevenagel condensation reaction followed by esterification of phenols, Baker–Venkataraman rearrangements, and concluding with demethylation. The structural properties of DHHPD were confirmed by ^1^H-NMR, ^13^C-NMR (Varian 500 MHz, Varian Inc., Palo Alto, CA, USA), and gas chromatography-mass spectrometry (Shimadzu GCMS-QP5050A Mass Spectrometer, Shimadzu, Kyoto, Japan). The obtained compound had a purity of more than 95%, verified through high-performance liquid chromatography. Details of the C97 (DHHPD) synthesis and chemical properties were reported by Leong et al. [12]. DHHPD was donated for use in this study by Leong and colleagues from IBS, UPM.

### 4.2. Experimental Animals, Drugs, and Chemicals

Adult male Institute for Cancer Research (ICR) mice (25–30 g, 3–4 weeks old) were acclimatized in the Animal Facility, Faculty of Medicine and Health Sciences, UPM for 7 days upon arrival at the facility. All mice used in the study were euthanized by cervical dislocation immediately after each experiment. Data were collected in a randomized, single-blinded, and controlled experimental design. The animal studies were approved by the Institutional Animal Care and Use Committee, UPM (R088/2017).

The vehicle used contains a mixture of Tween 20, DMSO, and distilled water at a ratio of 5:5:90. Acetylsalicylic acid (ASA) was used as the standard reference drug in all experiments. Except for 2,2,2-tribromoethanol (10 mL/kg, intraperitoneal), which was dissolved in physiological saline prior to anesthesia, all drugs and DHHPD were dissolved in vehicle prior to injection. ASA and DHHPD were administered intraperitoneally (i.p.), while carrageenan, histamine, serotonin hydrochloride, and bradykinin acetate salt solutions were administered through the intraplantar (i.pl.) route. Reagents and drugs were purchased from Sigma-Aldrich Chemie GmbH (Steinheim, Germany).

### 4.3. Dose Determination and Route of Administration

The DHHPD doses used in this study were selected based on the results obtained from a pilot experiment in our laboratory and the doses that are effective in inducing analgesia in the mouse models of induced nociception [15].

Aspirin was administered at 100 mg/kg (i.p.). The intraperitoneal route was chosen to minimize variation in results with respect to other drugs/compounds that were administered through the same route.

Furthermore, ASA administered at 100 mg/kg (i.p.) induces COX inhibition but does not produce gastric mucosal lesion by itself despite the 95% inhibition of prostaglandin generation [56].

DHHPD was administered via the parenteral route of administration (intraperitoneal) as this route produces the highest bioavailability of DHHPD in addition to avoiding the first-pass metabolism, which commonly occurs in enteral administration.

### 4.4. Acute Toxicity Study

An acute toxicity study was conducted as previously described [57]. A total of 30 ICR mice were segregated into five groups (*n* = 6) and fasted for 2 h before being administered with DHHPD (0.1, 0.3, 1, and 3 mg/kg, i.p.) or vehicle (10 mL/kg, i.p.). Following DHHPD administration, the mice were monitored for 3 h for any abnormal behavior and clinical signs, such as sedation, motor impairment, respiratory distress, and diarrhea. Observation was performed daily during working hours for 7 days. Any incidences of mortality were recorded. On day 8, the mice were sacrificed by cervical dislocation. A post mortem examination was carried out on each mouse.

### 4.5. Anti-Inflammatory Study

#### 4.5.1. Carrageenan-Induced Paw Edema Test

The carrageenan-induced paw edema test was performed according to Sulaiman et al. [58]. A total of 36 mice were allocated into six groups (*n* = 6) and the basal thickness (C_0_) of the right hind paw of each mouse was measured before administration of DHHPD (0.1, 0.3, 1, and 3 mg/kg, i.p.), ASA (100 mg/kg, i.p.), or vehicle (10 mL/kg, i.p.). After 30 min, 0.02 mL of carrageenan (1% *w*/*v*) was injected into the intraplantar region of the hind paw. A digital vernier caliper was used to measure the increase in paw thickness (*C*_*t*_) immediately after carrageenan injection (0 h) and then every hour for 5 h after carrageenan injection. Any increase in paw thickness was taken as an indicator of inflammation. Equation (1) was used to calculate the inhibition of inflammation:

(1) $$\mathrm{Percentage}\;\mathrm{reduction}\;\mathrm{of}\;\mathrm{edema}\;=\;\frac{\left[(C_{t}-\;C_{0})\;in\;control\;mice-(C_{t}-\;C_{0})\;in\;treated\;mice\right]}{\left(C_{t}-\;C_{0}\right)\;in\;control\;mice}\times \;100$$

where *C_t_* = average paw thickness after carageenan treatment at time *t* and *C*_0_ = average initial (basal) paw thickness for each group.

#### 4.5.2. Cotton Pellet-Induced Granuloma Test

The cotton pellet-induced granuloma test was performed in mice according to a method that was described previously with minor modifications [16,58]. Mice were divided into six groups of six mice each and acclimatized for 1 week. On day 1, four of the groups of mice were pretreated with four different doses of DHHPD (0.1, 0.3, 1, and 3 mg/kg, i.p.), the positive control group was pretreated with ASA (100 mg/kg, i.p.), and the negative control group was pretreated with vehicle (10 mL/kg, i.p.). Thirty minutes after pretreatment, the mice were anesthetized and a sterilized cotton pellet (10 ± 0.5 mg) was subcutaneously placed in the dorsal region of each mouse to induce granuloma formation. Following the surgical procedure, the treatments were given intraperitoneally once daily for seven consecutive days. On day 8, the mice were sacrificed and the cotton pellets were removed by dissection and dried at 60 °C overnight for determination of the final weight. The average weights for each group were determined and compared to the control group. The difference between the initial and final dry mass was taken as the weight of the granulomatous tissues formed.

### 4.6. Involvement of Histaminergic, Serotonergic and Bradykininergic Systems

#### 4.6.1. Histamine-Induced Paw Edema Test

The test was performed as previously described with slight modification [59,60]. A total of 18 mice were divided into three groups and the basal thickness of the right hind paw was determined before administration of any drugs. One group was administered DHHPD intraperitoneally at the highest dose (3 mg/kg), while the two control groups were given ASA (100 mg/kg, i.p.) or vehicle (10 mL/kg, i.p.). After 30 min, a histamine solution (100 µg/paw) was injected into the intraplantar region of the right hind paw. The increase in paw thickness was measured using a digital vernier caliper every 10 min for the first hour and then every hour until 5 h after the histamine injection. Changes in paw thickness were considered as a measure of inflammation and inflammation inhibition activity was calculated using Equation (1).

#### 4.6.2. Serotonin-Induced Paw Edema Test

The test was performed as previously described with slight modification [59,60]. A total of 18 mice were divided into three groups and the basal thickness of the right hind paw was determined before administration of any drug. One group of mice (*n* = 6) received an i.p. injection of DHHPD at the highest dose (3mg/kg) while the remaining two groups were given ASA (100 mg/kg, i.p.) or vehicle (10 mL/kg, i.p.). After 30 min, a serotonin solution (100 µg/paw) was injected into the intraplantar region of each mouse’s right hind paw. The increase in paw thickness was measured using a digital vernier caliper [61] every 10 min for the first hour and then every hour until 5 h after the serotonin injection. Changes in paw thickness were considered as a measure of inflammation and the percentage inhibition was calculated using Equation (1).

#### 4.6.3. Bradykinin-Induced Paw Edema Test

The test was performed as previously described with slight modification [59,60]. A total of 18 mice were divided into three groups (*n* = 6) and the basal thickness of the right hind paw was determined before the administration of any drug. Mice in one treatment group were given an i.p injection of DHHPD at the highest dose (3 mg/kg), while the remaining two groups were given ASA (100 mg/kg, i.p.) or vehicle (10 mL/kg, i.p.). After 30 min, a bradykinin solution (10 nmol/paw) was injected into the intraplantar region of each mouse’s right hind paw. The increase in paw thickness was measured using a digital vernier caliper every 10 min for the first hour and then every hour until 5 h after the bradykinin injection. Changes in paw thickness were considered as a measure of inflammation and the percentage inhibition was calculated using Equation (1).

## 5. Conclusions

DHHPD at 0.1, 0.3, 1, and 3 mg/kg (i.p.) was shown to exhibit anti-inflammatory activities in the carrageenan-, histamine-, bradykinin-, and serotonin-induced paw edema models by suppressing the synthesis and/or release of histamine, bradykinin, and serotonin, in addition to likely altering the activities of COX and NOS during the first and second phases of the carrageenan-induced paw edema test. The results observed in our in vivo anti-inflammatory study are in agreement with the results from in vitro models. Finally, DHHPD at all doses significantly suppressed the proliferative phase of granuloma formation. The effectiveness of DHHPD in reducing edema and granuloma formation may be attributed to its better bioavailability and solubility in the circulation compared to curcumin as a result of its functional hydroxyl groups. However, mechanistic studies evaluating the effects of DHHPD on other autacoid mediators of inflammation are warranted to elucidate the mechanisms underlying its anti-inflammatory activity. The chronic toxicity effect, probable mutagenic activities and hematology profiles post DHHPD treatment are needed to support the results from the acute toxicity study further.

## Figures and Tables

**Table 1:** Effects of 5-(3,4-dihydroxyphenyl)-3-hydroxy-1-(2-hydroxyphenyl)penta-2,4-dien-1-one (DHHPD) in the carrageenan-induced paw edema test. Each value represents the mean paw thickness ± standard error mean (S.E.M) in mm, (*n* = 6).

Paw Thickness in mm (% Inhibition)							
Group	Dose (mg/kg)	Time Interval (h)					
		0	1	2	3	4	5
**Vehicle**	-	2.60 ± 0.07	3.55 ± 0.10 ^d^	3.67 ± 0.09 ^d^	3.77 ± 0.07 ^d^	3.80 ± 0.07 ^d^	3.90 ± 0.03 ^d^
**DHHPD**	0.1	2.60 ± 0.07	3.44 ± 0.10 ^d^ (11.58%)	3.44 ± 0.04 ^d^ (21.50%) ^a^	3.35 ± 0.03 ^d^ (35.90%) ^b^	3.15 ± 0.07 ^d^ (54.16%) ^b^	3.05 ± 0.05 ^d^ (65.38%) ^b^
	0.3	2.59 ± 0.05	3.35 ± 0.02 ^d^ (20.00%) ^a^	3.26 ± 0.04 ^d^ (37.38%) ^b^	3.21 ± 0.02 ^d^ (47.00%) ^b^	3.06 ± 0.05 ^d^ (60.83%) ^b^	3.02 ± 0.04 ^d^ (66.92%) ^b^
	1	2.61 ± 0.04	3.32 ± 0.05 ^d^ (25.26%) ^a^	3.24 ± 0.03 ^d^ (41.12%) ^b^	3.14 ± 0.05 ^d^ (54.70%) ^b^	3.12 ± 0.03 ^d^ (57.50%) ^b^	2.95 ± 0.05 ^d^ (73.85%) ^b^
	3	2.60 ± 0.03	3.20 ± 0.02 ^d^ (36.84%) ^b^	3.05 ± 0.01 ^d^ (57.94%) ^b^	2.96 ± 0.03 ^d^ (69.23%) ^b^	2.86 ± 0.06 ^c^ (78.33%) ^b^	2.77 ± 0.02 (86.92%) ^b^
**ASA**	100	2.58 ± 0.05	3.37 ± 0.08 ^d^ (16.84%)	3.27 ± 0.02 ^d^ (35.51%) ^b^	3.16 ± 0.07 ^d^ (50.43%) ^b^	3.05 ± 0.02 ^d^ (60.83%) ^b^	2.98 ± 0.03 ^d^ (69.23%) ^b^

^a^*p* < 0.05 and ^b^ *p* < 0.0001 when compared to vehicle (two-way ANOVA followed by Dunnett’s post hoc test). ^c^ *p* < 0.01 and ^d^ *p* < 0.0001 when compared to 0 h (basal measurement) (two-way ANOVA followed by Dunnett’s post hoc test).

**Table 2:** Effect of DHHPD on granuloma tissue formation in mice. Each value is expressed as the mean weight of granuloma ± S.E.M in mg, (*n* = 6).

Group	Dose (mg/kg)	Granuloma Dry Weight (mg)	Inhibition (%)
**Vehicle**	-	66.84 ± 2.87	-
**DHHPD**	0.1	52.08 ± 1.82 ****	22.08
	0.3	45.07 ± 1.85 ****	32.57
	1	41.97 ± 1.45 ****	37.20
	3	33.92 ± 1.19 ****	49.25
**ASA**	100	33.62 ± 1.50 ****	49.70

**** *p* < 0.0001 when compared to vehicle (one-way ANOVA followed by Dunnett’s post hoc test). ASA: acetylsalicylic acid.
